# Inhibitory Effect of Honeysuckle (*Lonicera japonica* Thunb.) Extract on the Melanosis and Quality Deterioration of Pacific White Shrimp (*Litopenaeus vannamei*) During Cold Storage

**DOI:** 10.3390/foods14172928

**Published:** 2025-08-22

**Authors:** Ouyang Zheng, Huijie Chen, Xiaoye Jia, Yamei Liu, Qinxiu Sun, Zefu Wang, Shucheng Liu

**Affiliations:** 1 Guangdong Provincial Key Laboratory of Aquatic Product Processing and Safety, Guangdong Province Engineering Laboratory for Marine Biological Products, Guangdong Provincial Engineering Technology Research Center of Seafood, Key Laboratory of Advanced Processing of Aquatic Product of Guangdong Higher Education Institution, College of Food Science and Technology, Guangdong Ocean University, Zhanjiang 524088, China; zhengouyang07@163.com (O.Z.); 13433367511@163.com (H.C.); m13061594700@163.com (X.J.); 11211219mm@stu.gdou.edu.cn (Y.L.); sunqinxiugo@163.com (Q.S.); 2 Collaborative Innovation Center of Seafood Deep Processing, Dalian Polytechnic University, Dalian 116034, China

**Keywords:** *Lonicera japonica* Thunb. extract, shrimp melanosis, physicochemical quality, microorganism

## Abstract

The effect of *Lonicera japonica* Thunb. (LJT) extract on melanosis and quality deterioration of Pacific white shrimp (*Litopenaeus vannamei*) during cold storage was evaluated, using 4-hexylresorcinol (4-HR), a commercial melanosis inhibitor, as a positive control. The results showed that LJT inhibited polyphenol oxidase (PPO) activity and complexed copper ions in a dose-dependent manner (LJT < 16 mg/mL), indicating that LJT has the potential to inhibit shrimp melanization. Visual image and grayscale value analysis revealed that LJT and 4-HR effectively alleviated melanosis and delayed deterioration of shrimp quality stored at 4 °C, with LJT exhibiting a superior effect. LJT samples displayed a lower total number of colonies, total volatile base nitrogen value, and thiobarbituric reactive substance value than the others (*p* < 0.05), suggesting that LJT exerted the most potent inhibition on microbial growth and lipid oxidation in shrimps during cold storage. Consequently, LJT effectively retarded the melanosis and quality deterioration of shrimps.

## 1. Introduction

Pacific white shrimp (*Litopenaeus vannamei, L. vannamei*) is popular among consumers around the world because of its delicious flavor, high protein content, and rich nutritional value. It is one of the most widely cultivated shrimps, accounting for approximately 70% of the total shrimp production [1]. At the species level, *L. vannamei* is the species with the highest production in 2020, reaching 6.8 million tons [2]. Due to the substantial protein, water, and active endogenous enzymes within *L. vannamei*, it is highly perishable during storage [3]. In addition, phenolic compounds within its body are oxidized into quinones under the catalysis of polyphenol oxidase (PPO), further polymerizing with proteins, amino acids, and quinones to form dark brown substances, resulting in shrimp melanosis [4]. Shrimp melanosis drastically reduces its sensory appeal and economic value, weakening consumers’ desire to buy shrimps.

To address the melanosis issue, various methods and techniques have been employed. Researchers focus primarily on controlling the key components of enzymatic oxidation reactions, such as enzyme activity and oxygen content [5]. Sulfite is commonly employed to decelerate the rate of melanosis in shrimps and other crustaceans. However, the SO_2_ produced during the metabolism of sulfate food additives in the food residues of these substances induces allergic reactions in asthma patients and threatens human health [6]. In some countries or regions, the use of sulfite additives has even been banned. Accordingly, people are gradually seeking safer and more efficient inhibitor alternatives. Four-hexylresorcinol (4-HR) is a safe substitute for sulfite [7]. As a derivative of resorcinol, 4-HR can specifically bind to PPO and inhibit the enzymatic reaction, thereby achieving the purpose of inhibiting shrimp melanosis. Although 4-HR does not inflict obvious harm to human health, it is expensive and increases production costs. With the improvement in consumers’ understanding of food additives, the conditions they choose are becoming increasingly strict. Thus, identifying natural additives to replace chemical preservatives is necessary. Antioxidant-rich extracts from dual-purpose (medicinal and edible) plants exhibit specific binding affinity toward PPO, effectively blocking the initiation of melanosis in *L. vannamei* through competitive inhibition at the enzyme’s active site, thereby positioning these extracts as generally recognized as safe-compliant alternatives to synthetic sulfites in shrimp preservation [5]. In addition, plant-derived inhibitors have the advantages of being widely available, easy to operate and use, and low in price. In particular, substances derived from plant extracts using water as the medium have the characteristics of being easily soluble.

*Lonicera japonica* Thunb. (LJT) extract, a prototypical medicinal–edible dual-purpose botanical, has emerged as a multifunctional bioactive source with documented antioxidant, antimicrobial, and enzymatic inhibitory capacities [8]. The aqueous LJT extract manifests as a pale-yellow to greenish solution with a mild, subtly sweet taste profile, positioning it as an organoleptically compatible and safety-compliant candidate for crustacean melanosis inhibition [9]. Our preliminary comparative analysis of five antioxidative medicinal–edible extracts (including green coffee beans, Chinese prickly ash, chili pepper, and mango) demonstrated LJT’s superior anti-melanosis efficacy in *L. vannamei* (Unpublished). However, the mechanism of the inhibitory effect of LJT extract on shrimp melanosis is still unclear. Consequently, the inhibition effect of LJT on PPO and the complexation ability of copper ions were evaluated in this study. Additionally, the impact of LJT extract on shrimp melanosis during cold storage was assessed. Simultaneously, the relationship between microorganisms, total volatile base nitrogen (TVB-N), thiobarbituric acid reactive substances (TBARSs), and melanosis was investigated, expecting to find natural inhibitors of shrimp melanosis.

## 2. Materials and Methods

### 2.1. Chemicals

Brij-35, L-β-3,4-dihydroxylphenyl, and alanine (L-DOPA) were purchased from Sigma-Aldrich (St. Louis, MO, USA). 4-HR was purchased from Guangzhou Qiyun Biotechnology Co., Ltd. (Guangzhou, China). The LJT extract with water as a solvent was extracted by Xi’an Baitai Biotechnology Co., Ltd. (Xian, China). The extract was subsequently concentrated, dried, and crushed to yield a light yellow powder. The process and standards for making dry powder of honeysuckle (*Lonicera japonica* Thunb.) extract refer to the provisions of the China Group Standard T/CCCMHPIE1.11-2016 [10] and the Chinese national standard GB/T 43808-2024 [11]. A micro-malondialdehyde (MDA) assay kit was acquired from Beijing Soleibao Technology Co., Ltd. (Beijing, China). All the above reagents were analytically pure.

### 2.2. Shrimp Preparation

Live, non-pregnant white shrimps of uniform size (weighing approximately 25 ± 2 g, with a body length of 15 ± 1 cm), selected based on their commercial prevalence and stable postmortem biochemical profiles, were purchased from the Happy Ocean Aquatic Market in Zhanjiang, Guangdong, China. The shrimps were then transported to the laboratory using a polyethylene plastic bag filled with oxygen-filled seawater. Fresh shrimps of the same size and similar body colors were selected. They were anaesthetized to death by immersion in ice water after being rinsed three times with sterile water. The shrimps were then drained before further immersion treatments. All shrimps were randomly and equally divided into three groups of 40 shrimps each. In previous studies, it was determined that the optimal condition for impregnating shrimps with LJT was soaking in a 16 mg/mL LJT solution for 17 min (Unpublished). Considering the maximum amount of 4-HR allowed to be used, the remaining group of shrimps was soaked in 20 mg/mL 4-HR solution for 15 min at 4 °C in the ratio of 1:2 (*w*/*v*) [12]. The treated shrimps were drained for 3 min on a screen at 4 °C. The untreated portion of the shrimps served as the control samples. One shrimp was placed on a polystyrene tray, covered with plastic, and stored at 4 °C. A total of 10 shrimps were used in sampling for each melanosis measurement. Random samples were randomly drawn for continuous testing and analysis for 3 days each until day 9.

### 2.3. Extraction of PPO from the Cephalothoraxes of Shrimps

The cephalothoraxes of shrimps were separated and ground into powder using liquid nitrogen. The resultant powder was then placed in polyethylene bags and stored at −80 °C, ensuring it was used for polyphenol oxidase (PPO) extraction within a week. The method of extracting PPO from cephalothoraxes of shrimp powder refers to Nirmal and Benjakul [13]. The pH of the extracted PPO phosphate buffer was fine-tuned from 7.2 to 7.5 to preserve the PPO activity in the extracted shrimps. All procedures were conducted at 4 °C. The powder (100 g) was mixed with 300 mL of extracting buffer (0.05 mol/L sodium phosphate buffer, pH 7.5, containing 1.0 mol/L NaCl and 0.2% Brij 35, *w*/*v*). The mixture was stirred continuously for 30 min, accompanied by centrifugation at 8000× *g* in 4 °C for a duration of 30 min, utilizing a refrigerated centrifugal device (Avanti JXN-26, Beckmancoulter., Pasadena, CA, USA), leaving the supernatant in storage. To maximize the precipitation of PPO, solid ammonium sulfate was added to the supernatant to achieve 40% saturation, followed by a 30 min standing period. The precipitate was dispersed in 0.05 mol/L phosphate buffer (pH 7.5). The same buffer served as the dialysate for 12 h, and was subsequently changed at least three times. Following insoluble precipitation, the PPO solution supernatant was removed by centrifugation (4 °C, 4000× *g*), stored at 0–4 °C, and typically used within 12 h. SDS-PAGE was performed with 8% gel to determine the molecular weight of PPO.

### 2.4. Determination of PPO Inhibition

The inhibitory effect of PPO by LJT extract was studied with L-DOPA as the substrate. The LJT extract was dissolved in distilled water at concentrations of 4, 8, 12, 16, and 20 mg/mL. LJT extract solution (1 mL) and PPO extract (1 mL) were mixed to obtain the final extract concentrations, i.e., 2, 4, 6, 8, and 10 mg/mL, respectively. The mixture was incubated at 45 °C for 30 min, followed by the addition of 4 mL of phosphate buffer solution (pH 6.0). To initiate the reaction, 600 mL of L-DOPA solution pre-incubated at 45 °C (15 mmol/L) was added to the mixture. After a 3 min incubation at 45 °C, the absorbance of the mixture was measured at 475 nm using a UV-2550 spectrophotometer (Shimadzu Scientific Instruments Co., Ltd., Suzhou, China). Enzyme activity was determined based on the alteration in absorbance. The PPO inhibitory activity was quantified as a percentage inhibition, which was calculated as follows:

(1) $$\mathrm{Inhibition}\;(\%)\;=\;\frac{A-B}{A}\times 100$$

where *A* is the PPO activity of the control group, and *B* is the PPO activity of the treatment group after adding LJT.

### 2.5. Determination of Copper-Chelating Activity

Copper sulfate solution containing 10 mg/mL sodium nitrate with a concentration of 0.1 mol/L was prepared and set aside. The copper sulfate solution of 0.1 mol/L was diluted to standard solutions of 10^–2^, 10^–3^, 10^–4^, 10^–5^, and 10^–6^ mol/L with 10 mg/mL sodium nitrate in turn. The electrode potential E (in millivolts) of a series of copper standard solutions was measured with an ion meter (Lightning magnetic PXSJ-216 ion meter, Shanghai Yidian Scientific Instruments Co., Ltd., Shanghai, China), and the standard curve was plotted with E pair-lg Cu^2+^. Solutions of 1, 5, 10, 15, and 20 mg/mL LJT extract (containing 10^–3^ mol Cu^2+^ and 10 mg/mL sodium nitrate) were prepared, respectively. After stirring at 37 °C until the reaction reached equilibrium, an ion meter was used to measure the E value of the solution with varying concentrations. The coordination rate of Cu^2+^ was calculated according to the following equation:

(2) $$\mathrm{Copper}\;\mathrm{chelation}\;\mathrm{activity}\;(\%)\;=\left(1-\frac{A}{B}\right)\times 100$$

where *A* represents the remaining Cu^2+^ concentration, mol/L; *B* represents the concentration of total Cu^2+^, mol/L.

### 2.6. Melanosis Score

The severity of melanosis in the shrimp samples was evaluated every 3 days by a panel of ten trained assessors using a standardized 10-point scoring system adapted from established methodologies [14]. To ensure consistent evaluation, reference images of *Litopenaeus vannamei* displaying graded melanosis severity (with annotated scores) were provided as visual aids during assessments. The scoring criteria defined 0 as “no visible melanosis” and 10 as “extensive melanosis” (covering 80–100% of the shrimp surface).

### 2.7. Melanosis Evaluation

The melanosis degree of shrimps was evaluated by the method of Kimbuathong, Leelaphiwat, and Harnkarnsujarit [15]. The shrimp sample image was captured using a Huawei Glory V30PRO (OXF-AN10) mobile phone, professional mode (ISO: 400, S:1/4000) in a sterilized photo box with a closed white background, under controlled light intensity and the same intensity. Photographs were converted to black and white binary images using ImageJ software (version 1.52a, National Institutes of Health). Additionally, Image J was used to quantify the black part of the image to calculate the gray value of the shrimp, thereby facilitating the analysis of melanosis color changes. The melanosis degree was calculated according to the following formula:

(3) $$\mathrm{Melanosis}\;(\%)=\frac{B-A}{A}\times 100$$

where *A* is the gray value of the shrimp sample on day 0, and *B* is the actual gray value of the shrimp sample.

### 2.8. Total Viable Count (TVC) Analyses

TVC was determined according to the Chinese national standard GB4789.2-2022 (2022). Initially, the shrimps were decapitated, shelled, eviscerated, shredded, and crushed into muscle paste. The shrimp sample (5 g) was added to 45 mL of aseptic saline (NaCl, 0.85 g/100 mL), and then homogenized for 1 min at room temperature (25 °C) using a BM-400HP sterile bead homogenizer (Shanghai Chubai Laboratory equipment Co., Ltd., Shanghai, China). The homogenized solution was diluted in a decimal system with aseptic saline until three consecutive suitable gradients. Subsequently, 100 μL of diluent solution was placed on a preset aseptic plate count agar medium and spread evenly. TVC was determined by incubating at 30 °C for 48 h.

### 2.9. Determination of Total Volatile Base Nitrogen

TVB-N was determined according to the method of the Chinese standard GB5009. 228-2016 (2017) with slight modifications. The standard titration solution of hydrochloric acid with a concentration of 0.0100 mol/L was utilized as the calibration solution. The parameters of the Kjeldahl nitrogen analyzer (Vapodest 450, C. Gerhardt, Königswinter, Germany) were set as follows: the liquid volume of water and alkali solution was 0, the amount of boric acid receiving solution was 30 mL, and the distillation time was set to 3 min. The shrimps were beheaded, shelled, and stripped of lines, and the shrimp muscle was minced in a processor (Jiuyang JYL-C230, Joyoung Co., Ltd., Jinan, China). The shrimp sample (weighing 5 g to the nearest 0.01 g) was finely chopped and transferred into a 50 mL centrifuge tube, to which 37.5 mL of distilled water was subsequently added. The mixture was shaken for 30 s to ensure even blending. Subsequently, the mixture was transferred to a special digestion bottle for the automatic Kjeldahl nitrogen analyzer (Vapodest 450, C. Gerhardt, Königswinter, Germany), to which 0.4 g of magnesium oxide was added. The special digestion bottle was promptly linked to the still to determine the volatile base nitrogen content. The TVB-N contents were expressed as mg nitrogen per 100 g of shrimp, which was calculated according to the following formula:

(4) $$\mathrm{TVB}-N(mg/100g)=\frac{(V1-V2)\times c\times 14}{m}\times 100$$

where *V*_1_ indicates the volume of the standard titration solution of hydrochloric acid consumed by the test solution, mL; *V*_2_ represents the volume of the standard titration solution of hydrochloric acid consumed by the reagent blank, mL; *c* represents the concentration of the standardized titration solution of hydrochloric acid, mol/L; 14 is the mass of nitrogen equivalent to 1 hydrochloric acid [*c*(HCl) = 1.000 mol/L] standard titration solution, g/mol; *m* is the sample mass, g; and 100 is the conversion factor of the calculated result into milligrams per hundred grams, mg/100 g.

### 2.10. Determination of Thiobarbituric Acid Reactive Substances

TBARS values in shrimp muscle were measured according to the method of Shiekh [4]. The MDA content of shrimps was determined using an MDA content assay kit (Beijing Soleibao Technology Co., Ltd., Beijing, China), with *A*_600_ correction accounts for scattering from insoluble particles and chromogenic interferences (e.g., protein–lipid complexes). The MDA content was calculated according to the following formula:

(5) $$\mathrm{MDA}(nmol/g)=53.673\times \frac{\Delta A_{532}-\Delta A_{600}}{W}$$

where *∆A*_532_ = *∆A*_532 sample_-*∆A*_532 blank_, *∆A*_600_ = *∆A*_600 sample_-*∆A*_600 blank_; *∆A*_532 sample_ and *∆A*_600 sample_ are the absorbance of the sample at 532 nm and 600 nm, respectively; *∆A*_532 blank_ and *∆A*_600 blank_ are the absorbance of the blank at 532 and 600 nm, respectively; and *W* is the mass of the sample, *W* = 0.1 g.

### 2.11. Liquid Chromatography–Mass Spectrometry (LC-MS) Identification of LJT Extract

The components of LJT were analyzed using an Agilent 1200 HPLC system coupled with a SCIEX QTRAP^®^ 6500+ mass spectrometer (ESI source). Chromatographic separation utilized a Waters Xselect HSS T3 column (2.5 µm, 2.1 × 150 mm^2^) with a mobile phase of 0.1% formic acid (A: H_2_O; B: ACN) at 0.4 mL/min, 50 °C column temperature, and DAD detection at 280/352 nm. MS parameters included curtain gas (35 psi), ion source gases 1/2 (60 psi each), 550 °C temperature, and ion-spray voltage (±5500 V for positive/negative modes). Analysis employed multiple reaction monitoring (MRM) via a self-built database (novoDB). Quantitation relied on Q3 sub-ion peak areas, while qualitative identification combined Q1/Q3 ions, retention time (RT), declustering voltage (DP), and collision energy (CE). Data processing in SCIEX OS V1.4 applied the following thresholds: peak height ≥ 500, S/N ≥ 5, and 1 smoothing point. Metabolite identification/quantification was based on integrated sub-ion peak areas. Statistical analysis highlighted significant metabolic shifts, with pathway impacts validated against the literature.

### 2.12. Sensory Evaluation

The sensory evaluation protocol employed a rigorously trained panel of 15 assessors screened through ISO-aligned triangle tests (85% accuracy threshold) who evaluated cooked shrimp samples at days 0, 3, and 6 of refrigerated storage (post-day-6 samples excluded per sensory rejection thresholds) using a standardized 10-point scale across seven key attributes: appearance, odor, color, muscle morphology, texture, taste, and overall acceptability. The samples were prepared by boiling whole shrimp in water (1:4 *w*/*v* ratio) for precisely 4 min at 100 °C, and then immediately served under controlled lighting conditions (1000 lux daylight-mimicking bulbs) in partitioned booths to prevent cross-sample influence, with randomized coding and palate-cleansing procedures (mineral water) implemented between assessments to ensure unbiased scoring and methodological reproducibility consistent with international sensory analysis standards for aquatic products.

### 2.13. Statistical Analysis

All experiments were performed in triplicate, and the results are presented as the mean ± standard deviation. Data were analyzed by analysis of variance with Duncan’s multiple range test (*p* < 0.05) using SPSS (version 19.0, SPSS Inc., Chicago, IL, USA). A *t*-test was conducted for paired comparison. All figures were generated using Origin 2021 software (Origin Lab, Northampton, MA, USA). The correlation analysis and principal component analysis were performed using Origin 2021 software (Origin Lab, Northampton, MA, USA). The Pearson model was used for correlation analysis.

## 3. Results

### 3.1. Analysis of Composition Substances in LJT

LJT component analysis is vital for the preservation mechanism. Utilizing retention time, fragmentation patterns, and database comparisons, over 1800 compounds representing various species—including phenolic acids, flavonols and flavones, cinnamic acids and derivatives, and organic acids and derivatives, as well as benzoic acids and derivatives—were successfully identified. The principal active compounds are detailed in Table 1.

Targeted metabolomics results revealed that LJT extract was abundant in phenolic acids, flavonoids, cinnamic acids, and various other polyphenols. Specifically, phenolic acids constituted 18.2% of the relative content, and flavonols and flavonoids made up 29.0%. Additionally, cinnamic acids and their derivatives accounted for 0.5%. Organic acids and their derivatives represented 10.4%, and benzoic acids and their derivatives contributed 0.4%. Phenolic acids are regarded as one of the main active components of LJT, with chlorogenic acid serving as an important indicator for evaluating the quality of LJT extract. Phenolic acid is the primary active component of the LJT antibacterial agent, exhibiting enzyme inhibition properties, among which are caffeic acid and quinic acid [16]. 3,5-bis-O-caffeoyl quinic acid, 4,5-bis-O-caffeoylquinic acid, luteoloside, 3-O-caffeoylquinic acid, and secoxyloganin have been verified to have potent antibacterial and bactericidal activities [17]. In addition, compounds such as isochlorogenic acids A, B, and C, along with luteolin 7-O-glucopyranoside and other phenolic acids, have inhibitory effects on PPO activity [18,19]. Flavonoids in LJT, such as catechins (digallocatechin), kaempferol, rutin, and their derivatives, have antioxidant effects due to their rich phenolic hydroxyl groups. They have been reported to inhibit PPO and mitigate melanin formation in crustaceans during storage [20]. Catechins may be competitive inhibitors of PPO due to their structural resemblance to the PPO substrate L-DOPA. In addition, aromatic carboxylic acids of cinnamic acid and its derivatives, such as p-coumaric acid, ferulic acid, and sinapic acid, are also competitive inhibitors of PPO activity [21]. Nilesh et al. [21] believed that ferulic acid also inhibited the further formation of black compounds by reverting quinones back to diphenols or through interaction with dopachrome to form a light yellow complex. Benzoic acid has the function of inhibiting the production of melanin and killing bacteria. Small-molecule organic acids affect the growth rate of PPO active agent bacteria by changing the pH of the system. In general, these findings indicate that LJT extract contains melanosis-inhibitory and bacteriostatic active ingredients; therefore, LJT extract has the potential to be a melanosis inhibitor and preservative for *L. vannamei*.

### 3.2. PPO Inhibitory Activity by LJT Extract

**Figure 1:**
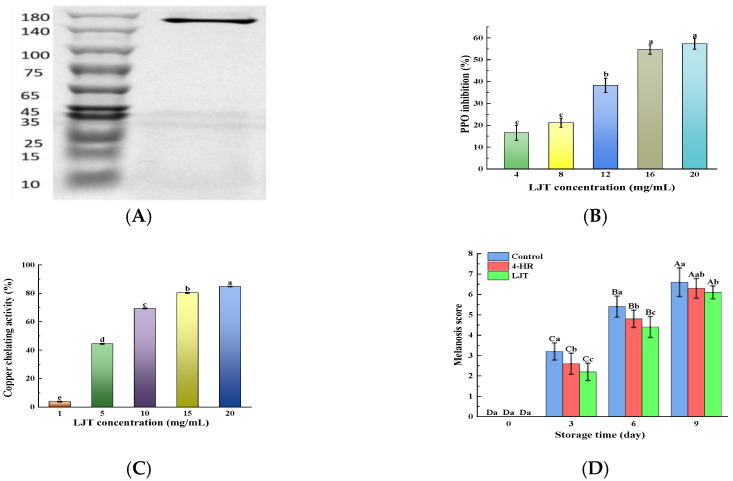
SDS-PAGE of PPO (**A**); PPO inhibition activity ((**B**), %); copper chelating activity ((**C**), %); and melanosis score (**D**) of various concentrations of LJT extract. Different lowercase letters in the same index indicate significant differences (*p* < 0.05), Different uppercase letters in the same index represent significant difference in different storage time in the same treatment group (*p* < 0.05). Bars in A represent the standard deviation (*n* = 10), others (*n* = 3).

Shrimp melanosis is commonly attributed to PPO catalytic oxidation activity. The rate of melanosis development in shrimps is influenced by PPO activity; as PPO activity increases, melanosis progresses more rapidly [5]. The crude PPO enzyme solution was verified and analyzed by SDS-PAGE and found to be a single band (Figure 1A) with a molecular weight of about 160 KD, indicating that PPO has successfully achieved purification. It was observed that the suppressive effect of LJT extract on PPO activity escalated alongside the growing concentration of the extract, indicating a dose-responsive pattern (Figure 1B). The enhancement of the inhibitory impact on PPO by LJT extract was significant when concentrations exceeded 8 mg/mL. Beyond 16 mg/mL, the inhibition of PPO activity by the LJT extract slowed down and even indicated that the inhibitory ability plateaued without further increase. The main components of LJT extract were identified as being notably rich in phenolic acids, flavonoids, cinnamic acids, benzoic acids, and other small-molecule organic acids in the present study (Table 1). These components have been proven to have PPO inhibitory properties, with chlorogenic acid, caffeoylquinic acid, rutin, quercetin, and luteolin being particularly notable [20,22]. There are three potential mechanisms through which LJT may inhibit PPO activity. Firstly, compounds rich in phenolic hydroxyl groups in LJT, such as phenolic acids, flavonoids, cinnamic acids, and benzoic acids, are recognized as primary inhibitors of PPO. These compounds compete with PPO substrates for interaction with the enzyme’s active sites, thereby suppressing its activity since the phenolic hydroxyl groups are crucial for PPO’s catalytic oxidation [23]. The hydroxyl group on the B ring of luteolin binds to Asn81 and Cys83, occupying the active site of PPO and competitively inhibiting PPO. In addition, luteolin destroys the tertiary structure of tyrosinase through hydrogen bonding, thereby inhibiting the activity of tyrosinase [24]. Phenolic compounds could interact with proteins or enzymes via a hydrogen bond or hydrophobic interaction [21]. A moderate increase in LJT concentration (16 mg/mL) increased the amount of phenolic hydroxyl group binding to PPO active sites, resulting in a rapid increase in PPO inhibition rate. However, as most enzyme active sites become occupied, further increases in LJT concentration lead to a more gradual rise in the inhibition rate. Secondly, the phenolic hydroxyl groups of phenolic derivatives and organic acids in LJT chelate with Cu^2+^ in the active center of PPO, effectively causing a loss of enzymatic activity [25]. The amount of phenolic derivatives and organic acids increases with a moderate increase in LJT concentration, resulting in an increase in Cu^2+^ chelation and PPO inhibition rate. As the level of Cu^2+^ chelation approached saturation, the further increase in extract (16–20 mg) resulted in a gradual change in PPO inhibition rate. Caffeic acid, tannin, and ferulic acid all exhibit the activity of chelating copper ions and are related to the polyhydroxylation structure [26]. Furthermore, certain researchers suggest that the phenolic acids, flavonoids, cinnamic acids, and benzoic acids present in LJT possess potent reducing properties. They convert quinone, the intermediate product of PPO activity, into phenols, thereby reducing the measured PPO activity [27]. Polyphenols inhibit PPO activity by donating electrons from their hydroxyl groups to quinone intermediates in dopa pigment synthesis, thereby reducing these intermediates back to dopa, and disrupting the enzymatic oxidation process [21]. The moderate increase in phenolic acids, flavonoids, cinnamic acids, and benzoic acids content in the system (LJT content 4–16 mg/mL) led to an increase in the amount of quinone reduced in the system, consequently enhancing the rate of PPO inhibition. However, as these compounds approached saturation levels in the system (with LJT content from 16 to 20 mg/mL), the rise in PPO inhibition rate became less pronounced. In summary, LJT extract was capable of effectively suppressing PPO activity in shrimps at a specific concentration range (LJT content < 16 mg/mL), showing a dose-dependent effect.

### 3.3. Copper-Chelating Activity by LJT Extract

Copper ions (Cu^2+^), serving as the active center of PPO, influence the activity of PPO [28]. Investigating the chelating activity of Cu^2+^ aids in elucidating the mechanism behind PPO inhibition and identifying inhibitors of PPO. The chelating activity of LJT initially rose sharply with increasing LJT concentration up to 15 mg/mL, then increased at a slower rate when the LJT concentration exceeded 15 mg/mL (Figure 1C). The Cu^2+^ chelating activity of LJT was consistent with its inhibition of PPO activity. This indicated that Cu^2+^ chelating is one of the mechanisms by which it inhibits the PPO of shrimp. The phenolic acids and flavonoids in LJT extract were abundant in ortho-hydroxyl groups. It is reported that plant-derived polyphenols exhibit Cu^2+^ chelating activity depending on the total number of ortho-hydroxyl groups present in the phenols [4,26]. These hydroxyl groups may play the role of antioxidants by complexing with metals with redox properties or as scavengers of free radicals [29]. As the concentration of LJT increased, the total number of o-hydroxyl groups also rose, enhancing its capacity to bind Cu^2+^. However, once Cu^2+^ was complexed by LJT, the free Cu^2+^ concentration diminished, impacting the binding efficiency of LJT. Overall, the LJT extract demonstrated effective Cu^2+^-chelating activity.

### 3.4. Changes in Shrimp Melanosis

**Figure 2:**
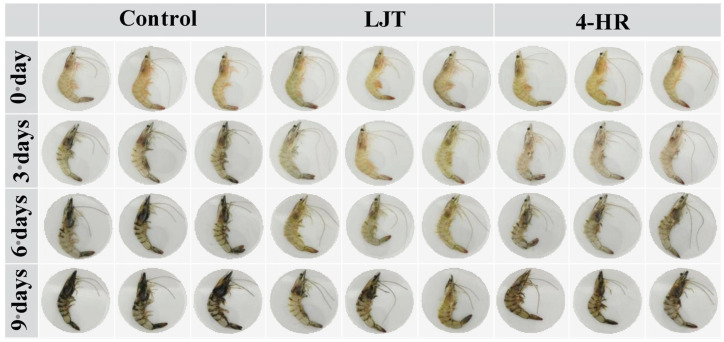
Photos of shrimps treated with different extracts during cold storage. Control: untreated shrimps; 4-HR: 4-hexylresorcinol at a concentration of 20 mg/mL; LJT: *Lonicera japonica* Thunb. at a concentration of 16 mg/mL. Sample shrimps were soaked for 15 min.

The visual changes in shrimps directly impact consumers’ willingness to purchase, serving as the most immediate indicator for assessing shrimp melanosis. As shown in Figure 2, at the initial stage of storage (day 0), all samples exhibited characteristic transparency, brightness, and greenish-yellow color. As the storage duration increased, the color of shrimps progressively exhibited melanosis. By the third day of storage, the control group exhibited blackening in the heads, tails, and shells, whereas the color of shrimps treated with 4-HR and LJT remained largely unchanged. Upon the sixth day of storage, black appeared in the head, tail, and back of the 4-HR and LJT groups, but the LJT group was significantly less blackened than the other two groups. By the 9th day, the color of the control group was the darkest, especially on the cephalothorax (head), tail, exoskeleton, and jointed leg. Only slight blackening was observed at the tail, articulated legs, cephalothorax, and exoskeleton of samples treated with the LJT extract. These results indicated that 4-HR and LJT inhibited shrimp melanosis, and LJT had a better inhibition effect than 4-HR.

The occurrence of shrimps exhibiting melanosis after death may be due to the activation of prophenoloxidase (proPPO) into PPO, which catalyzes the oxidation of tyrosine in shrimps to form quinone compounds, which further polymerize into black brown substances [4,30]. Due to the different distribution of PPO in shrimps, the highest levels of PPO were found in the head and thorax, followed by the tail and shell of shrimps [31]. Therefore, shrimp melanosis first occurred in the head and chest, where the melanosis was the most severe, followed by melanosis in the tail and shrimp shell, which was second in severity. The phenol hydroxyl group in 4-HR is identical to the PPO-catalyzed substrate tyrosine, mainly through competing for PPO catalytic sites, reducing the rate of PPO-catalyzed tyrosine, therefore inhibiting the occurrence of melanosis [32]. While LJT contains polyphenols and flavonoids, as well as cinnamic and benzoic acids, these compounds can competitively bind to PPO active sites and chelate active Cu^2+^ in PPO centers. Consequently, LJT reduces the intermediate product, quinone, of black spot disease through three pathways, thereby inhibiting PPO activity (Figure 1B) and preventing the occurrence of melanosis. A synergistic effect may have been exerted between phenolic acid, flavonoid, cinnamic acid, and benzoic acid compounds in the LJT extract, thereby enhancing PPO inhibitory activity [33]. These may be the reasons why LJT had a better inhibitory effect on melanosis than 4-HR.

**Figure 3:**
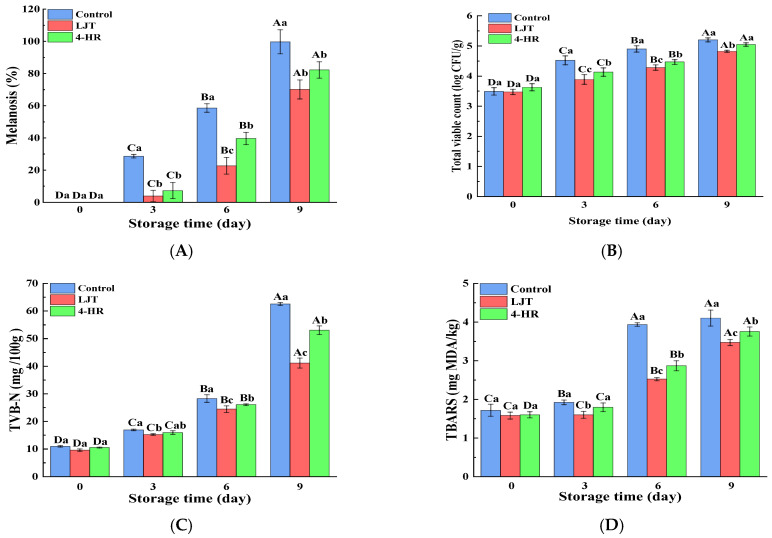
Alterations in melanosis ((**A**), %), TVB-N ((**B**), mg N/100 g), TBARSs ((**C**), mg MDA/kg), and total viable count ((**D**), log CFU/g) of the control samples and samples treated with LJT extract and 4-HR during storage. TVB-N, total volatile basic nitrogen; TBARSs, thiobarbituric acid reactive substances. Significant differences (*p* < 0.05) were found between means denoted by different uppercase letters within the same treatment group; significant differences (*p* < 0.05) were found between means denoted by different lowercase letters between different treatment groups on the same day.

Melanosis scores in shrimp samples treated with LJT (16 mg/mL) and 4-HR (20 mg/mL), compared to the control group, are summarized in Figure 1D. Initial analysis revealed no detectable melanosis (score = 0) in any group at day 0, indicating that no visible melanosis appeared and no differences in the shrimp samples with various treatments (*p* > 0.05). During refrigerated storage, melanosis severity progressively increased across all groups. The control group exhibited significantly higher melanosis indices than both treated groups throughout the study period (*p* < 0.05), with the 4-HR-treated samples showing intermediate values, and the LJT group demonstrating the lowest melanosis scores. The degree of melanosis in the area was quantified by image analysis (Figure 3A). Melanosis increased progressively with storage time in all samples (*p* < 0.05). This phenomenon may be attributed to the fact that with the increase in quinones formed by PPO catalytic oxidation, quinone substances further underwent condensation polymerization, resulting in a gradual increase in brown and melanosis substances. The accumulation of brown and melanosis substances promoted the blackening of shrimps [34]. Throughout the storage period, the melanosis levels were significantly lower in the LJT and 4-HR groups than in the control group (*p* < 0.05). The delayed development of melanosis formation in shrimps treated with LJT extract was probably associated with the high PPO inhibitory activity of LTJ extract (Figure 1B). This effect can be linked to the presence of polyphenols, including phenolic acids, flavonoids, cinnamic acids, and benzoic acids, in LJT extract, which suppressed the activity of PPO in shrimp. Byun et al. [35] also found that phenols in LJT were a promising PPO inhibitor. Consequently, phenolic acids, flavonoids, cinnamic acids, and benzoic acids in LJT extract synergistically inhibited PPO activity, thus delaying the occurrence of shrimp melanosis. As a derivative of resorcinol, the structure of 4-HR is similar to that of the PPO substrate. The hydroxyl group on the 4-HR benzene ring possesses the capacity to compete with the substrate for binding at the active site of the oxidase enzyme [36]. Therefore, 4-HR delayed melanosis in shrimps. Notably, no significant melanosis was observed in shrimp samples treated with LJT extract and 4-HR during 3 days of storage (*p* > 0.05). However, by the sixth day, the melanosis of the LJT group had significantly less melanin deposition than the 4-HR group (*p* < 0.05), suggesting that LJT extract was more effective than 4-HR in inhibiting melanosis. It has been reported that 4-HR binds to the active catalytic sites of PPO to form stable compounds [32]. Through the continuous consumption of PPO, the contact concentration between enzyme and substrate was reduced, which in turn delayed the oxidation of phenols catalyzed by PPO, thereby producing blackening substances at a slower rate. Therefore, 4-HR inhibited the melanosis of shrimps. The primary constituents of LJT include phenolic acids, flavonoids, cinnamic acids, and benzoic acids, which have been documented to exhibit strong PPO inhibitory properties [37]. Phenolic acids not only bind to the centrally active Cu^2+^ of PPO but also interact with various amino acids within PPO to form additional hydrogen bonds. Phenolic acid binding to the hydrophobic cavity of polyphenol oxidase leads to weakened hydrophobic interactions of PPO [25]. The phenolic acid not only avoided the entry of the substrate by occupying the catalytic center, but also induced a conformational change in PPO, effectively reducing or even inactivating the PPO activity. Moreover, the polyphenols in LJT exhibited strong antioxidant properties, which may inhibit melanosis by decreasing the production of melanosis precursors, quinones. Therefore, LJT demonstrated superior melanosis inhibition compared to 4-HR. Overall, LJT exhibited a strong capacity to suppress melanosis in shrimps.

### 3.5. Changes in the Microbiology of Shrimp

The total viable count (TVC) has a vital role in the deterioration of shrimp quality during storage. As depicted in Figure 3B, the initial value of TVC in fresh shrimps was 3.49 log CFU/g on day 0, which indicates high quality levels and aligns closely with the findings of numerous researchers [4,19,38]. The total number of initial TVC was related to the culture environment of live shrimps, which reflected the number of colonies in culture waters. The TVC of all shrimps exhibited a significant increase as storage time lengthened (*p* < 0.05). The abundant soluble protein and water content in shrimp muscle created an environment conducive to the swift proliferation of microorganisms, leading to rapid growth and reproduction of microorganisms within shrimp muscle [39]. Therefore, the microorganisms in shrimp muscle tended to rise progressively as storage time elapsed. Throughout the entire storage period, the TVC for samples treated with LJT and 4-HR remained consistently below that of the control group (*p* < 0.05), with the LJT-treated group exhibiting a lower TVC than those treated with 4-HR (*p* < 0.05). This indicates that both LJT and 4-HR possess antibacterial properties, and LJT demonstrated superior antibacterial efficacy compared to 4-HR. The inhibitory mechanism of LJT extract on microorganisms could be due to the collaborative antibacterial impact from the organic acids and flavonoids present in LJT [40]. This study revealed that LJT was abundant in organic acids and flavonoids (Table 1). Specifically, chlorogenic acid and its derivatives form the predominant organic acid fraction within LJT, which have been demonstrated to impede microbial growth and reproduction by causing damage to microbial cell membranes [17]. Furthermore, the main flavonoids found in LJT, namely luteolin and its derivatives, have been demonstrated to modify membrane permeability and suppress the synthesis of soluble proteins and nucleic acids in bacteria, thereby inducing microbial death [41]. Additionally, Punia et al. [42] discovered that the primary flavonoids in LJT exhibit broad-spectrum antibacterial properties. Therefore, the LJT extract effectively suppressed microbial growth during the cold storage of shrimp. However, 4-HR, a polyphenolic compound, induced microbial death by disrupting membrane permeability and integrity [43]. Consequently, the antibacterial impact of 4-HR was marginally less potent than that of LJT. In conclusion, the LJT extract holds promise as a preservative for Pacific white shrimp.

### 3.6. Changes in the TVB-N of Shrimp

As an important indicator of freshness and quality of shrimp, TVB-N refers to the alkaline nitrogen-containing substances, including ammonia and amines, which are produced by protein breakdown during spoilage of animal food due to enzymes and bacteria [44,45]. As depicted in Figure 3C, the TVB-N values of all groups exhibited a significant increase corresponding to the duration of storage. A higher content of TVB-N signified that more amino acids, particularly methionine and tyrosine, had been degraded. Concurrently, a high TVB-N content indicates a more significant nutritional loss in shrimp muscle. The TVB-N value’s elevation could be due to the growing number of microorganisms in shrimps as storage duration lengthens, which led to an increase in the production of volatile alkaline nitrogen compounds, such as trimethylamine, dimethylamine, ammonia, and tertiary amines, resulting from enzymatic protein degradation in shrimps [44]. It is worth noting that the rate of increase in TVB-N content varied among the three treatments, with the control sample having the highest TVB-N content, followed by that of the 4-HR sample, while the LJT sample had the lowest TVB-N content during the entire storage process (*p* < 0.05). This may be attributed to the fact that LJT and 4-HR suppressed microbial growth and reproduction [46], reducing the production and accumulation of TVB-N. The antibacterial effect of LJT inhibition was superior to that of 4-HR (Figure 3B), resulting in a lower TVB-N content and better preservation of shrimp freshness in the LJT group.

### 3.7. Changes in the TBARSs of Shrimp

The oxidative rancidity of lipids is one of the primary causes of the deterioration of seafood flavor products and a significant factor influencing the quality of aquatic products. The TBARS value, a widely recognized assessment of lipid oxidation, gauges the extent of lipid oxidation by measuring the secondary product malondialdehyde [47]. As illustrated in Figure 3D, initial TBARS values for all samples fell within a narrow range of 1.60 to 1.72 mg MDA/kg, with no significant differences observed among the samples (*p* > 0.05). This is because at the onset of storage, the shrimps had just expired and had not yet experienced significant oxidation. Consequently, there was no pronounced difference in TBARS values between the treatment groups [48]. The TBARS value of all shrimps increased gradually with increasing storage time. There are numerous factors contributing to the rise in TBARS value in shrimps during storage. On the one hand, with longer storage time, the microbial content increased, accelerating metabolic decomposition that damaged shrimp tissue membranes and promoted the oxidation of unsaturated fatty acid esters within them [49]. Additionally, deterioration of shrimp tissue membranes facilitated the release of intracellular pro-oxidants, such as metal ions and lipoxygenase, which in turn accelerated lipid oxidation and consequently increased the TBARS value [7]. On the other hand, with increasing storage time, the proliferation and quantity of psychrophilic bacteria significantly escalated. These cold-loving bacteria produced lipase and phospholipase, leading to an increase in easily oxidized free fatty acids, resulting in an increase in TBARS values [50].

It is noteworthy that the control group showed the highest growth rate in TBARS values, followed by the 4-HR treatment group, and the LJT extract exhibited the lowest growth rate. During the initial three days, the TBARS content in the control and 4-HR treatment groups experienced a slight increase (*p* < 0.05), but no significant rise (*p* > 0.05) was found in the LJT treatment group. By day 6, TBARS levels were significantly lower in both the LJT and 4-HR groups than in the control group (*p* < 0.05). By the end of the 9 days of storage, the TBARS levels in all experimental groups remained below the maximum allowable content of 5 mg MDA/kg for marine products [51]. There are numerous reasons why LJT inhibits the increase in TBARS values. On the one hand, the phenolic acids and flavonoids in LJT possess potent antioxidant properties, which effectively hinder the initiation of lipid oxidation [52]. On the other hand, LJT also acts to suppress microbial growth, thereby diminishing fat oxidation that is typically induced by microbial activity, resulting in a reduction in TBARS values. In addition, the delayed lipid oxidation in LJT-treated shrimps may be attributed to the metal chelation of LJT compounds with metal chelation properties, which inhibited lipid oxidation induced by metal ions and suppressed the increase in TBARS values [53]. As a novel polyphenol antioxidant, 4-HR has the effect of delaying fat oxidation and inhibiting the growth of TBARS. However, the inhibitory effect of 4-HR on microbial proliferation, a key factor in fat oxidation, is not as effective as that of LJT. This may primarily explain why 4-HR was less effective in suppressing the growth of the TBARS value compared to LJT. Consequently, the LJT extract impeded lipid oxidation and prevented the rancidity of shrimp muscle, indicating that the LJT extract has promising use as a natural antioxidant.

### 3.8. Sensory Analysis

Sensory profiling revealed temporally progressive quality deterioration across all groups, with initial equivalence at day 0 (all attributes > 9.13, *p* > 0.05, Table 2) confirming treatment neutrality on fresh shrimp quality. By day 3, both LJT and 4-HR treatments demonstrated significant protective effects (*p* < 0.05) versus the controls in appearance, odor, and color retention, while LJT specifically outperformed 4-HR in texture preservation (8.33 vs. 8.07) due to enhanced muscle integrity. This divergence amplified by day 6, where LJT-treated shrimps maintained superior texture (7.80), taste (7.60), and overall acceptability (7.27) compared to 4-HR (7.07, 6.47, and 6.80 respectively; *p* < 0.05), correlating strongly with concurrent physicochemical measurements that showed LJT’s synergistic inhibition of protein degradation (lower TVB-N), lipid oxidation (reduced TBARSs), and microbial growth (suppressed TVC), which collectively delayed organoleptic failure by preserving structural proteins and lipid membranes while minimizing melanosis-induced visual defects, thereby extending sensory shelf-life by approximately 3 days versus conventional treatments.

### 3.9. Principal Component Analyses

**Figure 4:**
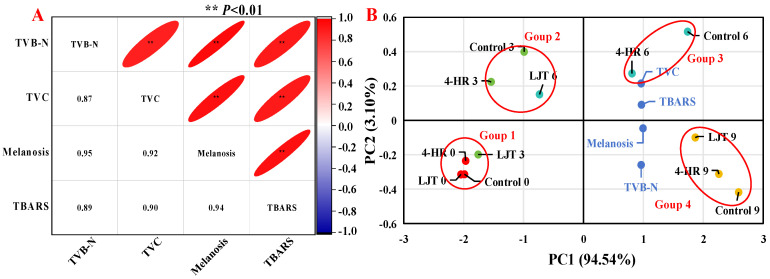
Correlation analysis (**A**) and principal component analysis (**B**) of freshness indexes in different treatments.

As illustrated in Figure 4A, melanosis had an extremely significant positive correlation with TVC, TVB-N, and TBARS values. The correlation coefficients of melanosis with TVB-N and TBARS values were 0.95 and 0.94, respectively, which were higher than that of TVC with 0.88. Correlation analysis showed that melanosis, TVC, TVB-N, and TBARSs exerted mutually reinforcing effects. A high degree of statistical similarity existed between TVC and melanosis, and TVB-N values were also found by many researchers [15,50,54].

For the principal component analysis, the variance contribution rate of the first principal component (PC1) was 94.54%, and that of the second principal component (PC2) was 3.10%, totaling 97.64%, and the relative eigenvalues were 3.78 and 0.12, respectively (Figure 4B). Thus, information on the principal component reflecting index was relatively complete. This analysis revealed significant differences among various treatment groups in different indicators. The TVB-N and TBARS values were close to the melanosis values, indicating a close relationship between them, which corresponds to the results of the correlation analysis (Figure 4A). In the direction of PC1, melanosis, TVC, TVB-N, and TBARS values showed a positive correlation. From the perspective of shrimp muscle quality, the effect of the immersion conditions on shrimps was significant, as there were significant differences in muscle quality between samples stored for the same time in the control, 4-HR, and LJT groups, indicating that these changes were largely attributed to the immersion conditions. The score plots of principal components (Figure 4B) show significant differences between shrimps soaked under different conditions and stored for different periods of time. Throughout the entire storage period, all samples could be roughly separated into four groups: The first group included LJT-0, LJT-3, control-0, and 4-HR-0; these were the samples with the lowest melanosis, TVC, TVB-N, and TBARS values. The second group included LJT-6, control-3, and 4HR-3 samples with relatively lower melanosis, TVC, TVB-N, and TBARS values. The third group included 4-HR-6 and control-6 samples with relatively higher melanosis, TVC, TVB-N, and TBARS values. And the fourth group included LJT-9, 4-HR-9, and control-9, which had the highest melanosis, TVC, TVB-N, and TBARS values. According to these results, LJT had the greatest impact on the preservation of the quality of shrimps during refrigeration, mainly improving the quality of shrimps from 0 to 3 d and 3 to 6 d, with less impact from 6 to 9 d. This further proved that LJT is an effective substance for inhibiting melanosis and freshness quality decline in shrimps during cold storage.

## 4. Conclusions

The effect of LJT on the quality changes in Pacific white shrimp during cold storage was investigated in the present study. The results indicate that LJT effectively inhibited PPO activity and chelated Cu^2+^ in a dose-dependent manner. The findings indicate that LJT had the potential to inhibit shrimp melanization, as demonstrated by the LJT group having smaller grayscale values and a brighter appearance compared to the 4-HR group. Compared with 4-HR, LJT had better antibacterial ability, thus a better ability to maintain the freshness of shrimp, as evidenced by its lower TVC and TVB-N values compared to 4-HR. In addition, LJT extract inhibited lipid oxidation in shrimps during cold storage, showing the lowest TBARS value among all groups. In general, LJT is a potential preservative for shrimps, which has the ability to inhibit the melanosis, spoilage, and oxidation of shrimps during cold storage. Further explanation of the pathway of the main active components of LJT to inhibit melanosis will be more conducive to revealing its mechanism of melanosis inhibition, and promote the application of LJT to inhibit shrimp melanosis.

## Figures and Tables

**Table 1:** The main active compounds in the LJT extract.

NO.	Identified Compound	Formula	M/W	RT	RC%	Cat
1	1-Caffeoylquinic acid	C_16_H_18_O_9_	354.095	5.46	4.37	**Phenolic acids**
2	Cryptochlorogenic acid	C_16_H_18_O_9_	354.309	5.44	1.88	
3	Isochlorogenic acid C	C_25_H_24_O_12_	516.451	7.05	1.62	
4	Chlorogenic acid	C_16_H_18_O_9_	354.31	5.40	1.59	
5	Quinic acid	C_7_H_12_O_6_	192.17	1.00	1.46	
6	Neochlorogenic acid	C_16_H_18_O_9_	354.309	4.879	1.43	
7	Isochlorogenic acid B	C_25_H_24_O_12_	516.451	6.77	1.26	
8	4,5-DCQA isochlorogenic acid C	C_25_H_24_O_12_	516.458	6.93	1.03	
9	Isochlorogenic acid A	C_25_H_24_O_12_	516.451	6.91	0.78	
10	1,5-Dicaffeoylquinic acid	C_25_H_24_O_12_	516.458	6.89	0.70	
11	Cynarin	C_25_H_24_O_12_	516.458	6.89	0.66	
12	Peonidin O-hexoside	C_22_H_23_O_11_	463.123	6.80	0.61	
13	1-O-Gentisoyl-D-glucoside	C_13_H_16_O_9_	316.079	4.56	0.28	
14	3-O-p-Coumaroylquinic acid	C_16_H_18_O_8_	338.1	5.92	0.25	
15	Caffeic acid	C_9_H_8_O_4_	180.16	5.80	0.13	
16	Protocatechuic acid	C_7_H_6_O_4_	154.12	4.57	0.06	
17	4-O-p-Coumaroylquinic acid	C_16_H_18_O_8_	338.1	5.93	0.04	
18	1-O-Feruloyl quinic acid	C_17_H_20_O_9_	368.1	6.23	0.03	
19	Catechol	C_6_H_6_O_2_	110.11	5.29	0.02	
20	3,4-Dihydroxybenzaldehyde	C_7_H_6_O_3_	138.12	5.31	0.01	
21	Ferulic acid	C_10_H_10_O_4_	194.19	6.88	0.01	
23	Kaempferol-3-O-rutinoside	C_27_H_30_O_15_	594.526	6.51	2.14	**Flavonols and flavonols**
24	Cyanidin O-rutinoside	C_27_H_31_O_15_	595.52	6.66	2.02	
25	Lonicerin	C_27_H_30_O_15_	594.526	6.73	1.97	
26	Luteolin 7-O-glucoside	C_21_H_20_O_11_	448.38	6.66	1.80	
27	Kaempferol7-O-beta-D-glucopyranoside	C_21_H_20_O_11_	448.377	6.65	1.71	
28	D-Pyroglutamic acid	C_5_H_7_NO_3_	129.114	1.62	1.64	
29	Kaempferol 3-O-robinobioside	C_27_H_30_O_15_	594.159	6.55	1.62	
30	Trifolin	C_21_H_20_O_11_	448.101	6.62	1.55	
31	Chrysoeriol 7-O-hexoside	C_22_H_22_O_11_	448.4	7.20	1.55	
32	Luteolin-4′-O-glucoside	C_21_H_20_O_11_	448.101	6.80	1.48	
33	Homoplantaginin	C_22_H_22_O_11_	462.403	7.19	1.42	
34	Chrysoeriol 7-O-rutinoside	C_28_H_32_O_15_	608.545	6.94	1.37	
35	Chrysoeriol 5-O-hexoside	C_22_H_22_O_11_	462.404	7.18	1.34	
36	Sophoricoside	C_21_H_20_O_10_	432.38	7.04	1.16	
37	Neodiosmin	C_28_H_32_O_15_	608.545	7.12	1.07	
38	Resokaempferol 7-O-hexoside	C_21_H_20_O_10_	434.4	7.08	0.91	
39	Rhoifolin	C_27_H_30_O_14_	578.52	6.94	0.91	**Flavonols and flavonols**
40	Isorhoifolin	C_27_H_30_O_14_	578.52	6.84	0.88	
41	Rutin	C_27_H_30_O_16_	610.518	6.45	0.61	
42	Kaempferol-3-gentiobioside	C_27_H_30_O_16_	610.525	6.28	0.36	
43	Gallocatechin-gallocatechin	C_30_H_26_O_14_	610.2	6.24	0.25	
44	Chrysoeriol O-hexosyl-O-pentoside	C_27_H_30_O_15_	594.526	7.10	0.22	
45	Chalcone	C_15_H_12_O	208.255	6.47	0.22	
46	Tricin 5-O-hexoside	C_23_H_24_O_12_	492.43	7.19	0.18	
47	Apigenin O-hexosyl-O-pentoside	C_26_H_28_O_14_	564.1	6.83	0.14	
48	Diosmetin	C_16_H_12_O_6_	300.26	8.91	0.13	
49	Luteolin	C_15_H_10_O_6_	286.24	8.06	0.13	
50	Tricin 7-O-hexoside	C_23_H_24_O_12_	492.436	7.63	0.10	
51	Malvidin 3-O-galactoside	C_23_H_25_O_12_	493	7.63	0.07	
52	Delphinidin 3-O-rutinoside	C_27_H_31_O_16_	611.5	6.35	0.05	
53	P-Hydroxy-cinnamic acid	C_9_H_8_O_3_	164.16	6.53	0.10	
54	trans-3,5-dimethoxy-4-hydroxycinnamaldehyde	C_11_H_12_O_4_	208.211	6.49	0.05	**Cinnamic acids and derivatives**
55	2-Hydroxycinnamate	C_9_H_8_O_3_	164.047	6.48	0.05	
56	p-coumaric acid	C_9_H_8_O_3_	164.16	6.55	0.05	
57	Ferulaldehyde	C_10_H_10_O_3_	178.185	7.53	0.04	
58	2-Methylcinnamic acid	C_10_H_10_O_2_	162.185	5.53	0.04	
59	Hydroxycinnamate	C_9_H_8_O_3_	164.158	5.54	0.03	
60	Cinnamic acid	C_9_H_8_O_2_	148.16	0.87	0.03	
61	Isoacteoside	C_29_H_36_O_15_	624.595	6.44	0.03	
62	Caffeic acid O-glucoside	C_15_H_18_O_9_	342	5.38	0.02	
63	Sinapinic acid	C_11_H_12_O_5_	224.21	6.87	0.02	
64	3,5-Dihydroxybenzoic acid	C_7_H_6_O_4_	154.12	4.46	0.21	**Benzoic acids and derivatives**
65	2,6-Dihydroxybenzoic acid	C_7_H_6_O_4_	154.12	6.13	0.07	
66	Terephthalic acid	C_8_H_6_O_4_	166.13	5.80	0.04	
67	Dibutyl phthalate	C_16_H_22_O_4_	278.152	13.42	0.04	
68	Gallic acid O-hexoside	C_13_H_16_O_10_	332	4.13	0.03	
69	2,3-Dihydroxybenzoic acid	C_7_H_6_O_4_	154.027	6.21	0.03	
70	Succinic acid	C_4_H_6_O_4_	118.09	1.94	2.63	**Other small molecule organic acid and its derivatives**
71	Citric acid	C_6_H_8_O_7_	192.12	1.52	1.45	
72	L(-)-Malic acid	C_4_H_6_O_5_	134.022	1.08	1.29	
73	Isocitrate	C_6_H_8_O_7_	192.124	1.49	1.24	
74	Methylmalonate	C_4_H_6_O_4_	118.09	1.94	1.09	
75	D-threo-isocitric acid	C_6_H_8_O_7_	192.124	1.41	0.48	
76	D-Xylonic acid	C_5_H_10_O_6_	166.048	0.9	0.47	
77	Lactic acid	C_3_H_6_O_3_	90.078	1.48	0.26	
78	Punicic acid	C_18_H_30_O_2_	278.3	12.65	0.20	
79	Glutaric acid	C_5_H_8_O_4_	132.115	0.70	0.16	
80	Dimethylmalonic acid	C_5_H_8_O_4_	132.042	0.67	0.16	
81	Oxalacetic acid	C_4_H_4_O_5_	132.07	0.69	0.14	
82	D-Galactonic acid	C_6_H_12_O_7_	196.155	0.92	0.12	
83	4-Hydroxy-2-oxoglutaric Acid	C_5_H_6_O_6_	162.098	0.70	0.12	
84	N-(2-Hydroxyethyl)iminodiacetic acid	C_6_H_11_NO_5_	177.155	1.02	0.11	

M/W: molecular weight (g/mol); RT: retention time (min); and RC: relative content (%).

**Table 2:** Sensory score of the control samples and samples treated with LJT extract and 4-HR during storage.

Storage Time (d)	Sample	Appearance	Odor	Color	Muscle Morphology	Texture	Taste	Overall
0	Control	9.87 ± 0.13 ^Aa^	9.87 ± 0.13 ^Aa^	10.00 ± 0.00 ^Aa^	10.00 ± 0.00 ^Aa^	10.00 ± 0.00 ^Aa^	9.53 ± 0.27 ^Aa^	10.00 ± 0.00 ^Aa^
	LJT	10.00 ± 0.00 ^Aa^	10.00 ± 0.00 ^Aa^	10.00 ± 0.00 ^Aa^	10.00 ± 0.00 ^Aa^	10.00 ± 0.00 ^Aa^	9.27 ± 0.27 ^Aa^	10.00 ± 0.00 ^Aa^
	4-HR	10.00 ± 0.00 ^Aa^	10.00 ± 0.00 ^Aa^	10.00 ± 0.00 ^Aa^	9.67 ± 0.33 ^Aa^	9.87 ± 0.07 ^Aa^	9.13 ± 0.59 ^Aa^	10.00 ± 0.00 ^Aa^
3	Control	7.20 ± 0.31 ^Bb^	7.53 ± 0.37 ^Bb^	6.53 ± 0.07 ^Bb^	8.53 ± 0.07 ^Ba^	7.27 ± 0.35 ^Bb^	7.87 ± 0.13 ^Ba^	6.60 ± 0.31 ^Bb^
	LJT	8.53 ± 0.07 ^Ba^	8.80 ± 0.12 ^Ba^	7.67 ± 0.18 ^Ba^	8.27 ± 0.13 ^Ba^	8.33 ± 0.18 ^Ba^	8.13 ± 0.18 ^Ba^	8.13 ± 0.13 ^Ba^
	4-HR	8.67 ± 0.13 ^Ba^	8.47 ± 0.18 ^Ba^	7.93 ± 0.13 ^Ba^	8.20 ± 0.20 ^Ba^	8.07 ± 0.68 ^Bab^	8.00 ± 0.20 ^Ba^	8.13 ± 0.13 ^Ba^
6	Control	6.27 ± 0.24 ^Cb^	7.07 ± 0.52 ^Ba^	5.53 ± 0.18 ^Bb^	7.13 ± 0.48 ^Cb^	6.73 ± 0.24 ^Bb^	6.80 ± 0.35 ^Cab^	6.33 ± 0.18 ^Bb^
	LJT	6.87 ± 0.33 ^Cab^	7.47 ± 0.29 ^Ca^	6.80 ± 0.35 ^Ba^	7.87 ± 0.07 ^Ba^	7.80 ± 0.20 ^Ba^	7.60 ± 0.31 ^Ba^	7.27 ± 0.18 ^Ca^
	4-HR	7.20 ± 0.42 ^Ca^	7.53 ± 0.29 ^Ca^	6.93 ± 0.29 ^Ba^	7.60 ± 0.12 ^Bab^	7.07 ± 0.27 ^Cab^	6.47 ± 0.24 ^Cb^	6.80 ± 0.46 ^Cab^

Different uppercase letters represent significant difference between different storage times for the same treatment (*p* < 0.05), and different lowercase letters represent significant difference between different treatment groups for the same storage time (*p* < 0.05).

## Data Availability

The original contributions presented in this study are included in the article. Further inquiries can be directed to the corresponding authors.
